# Precise Doppler shift compensation in the hipposiderid bat, *Hipposideros armiger*

**DOI:** 10.1038/s41598-018-22880-y

**Published:** 2018-03-15

**Authors:** Diana Schoeppler, Hans-Ulrich Schnitzler, Annette Denzinger

**Affiliations:** Animal Physiology, Institute for Neurobiology, University of Tübingen, Tübingen, Germany

## Abstract

Bats of the Rhinolophidae and Hipposideridae families, and *Pteronotus parnellii*, compensate for Doppler shifts generated by their own flight movement. They adjust their call frequency such that the frequency of echoes coming from ahead fall in a specialized frequency range of the hearing system, the auditory fovea, to evaluate amplitude and frequency modulations in echoes from fluttering prey. Some studies in hipposiderids have suggested a less sophisticated or incomplete Doppler shift compensation. To investigate the precision of Doppler shift compensation in *Hipposideros armiger*, we recorded the echolocation and flight behaviour of bats flying to a grid, reconstructed the flight path, measured the flight speed, calculated the echo frequency, and compared it with the resting frequency prior to each flight. Within each flight, the average echo frequency was kept constant with a standard deviation of 110 Hz, independent of the flight speed. The resting and reference frequency were coupled with an offset of 80 Hz; however, they varied slightly from flight to flight. The precision of Doppler shift compensation and the offset were similar to that seen in Rhinolophidae and *P*. *parnellii*. The described frequency variations may explain why it has been assumed that Doppler shift compensation in hipposiderids is incomplete.

## Introduction

**Figure 1:**
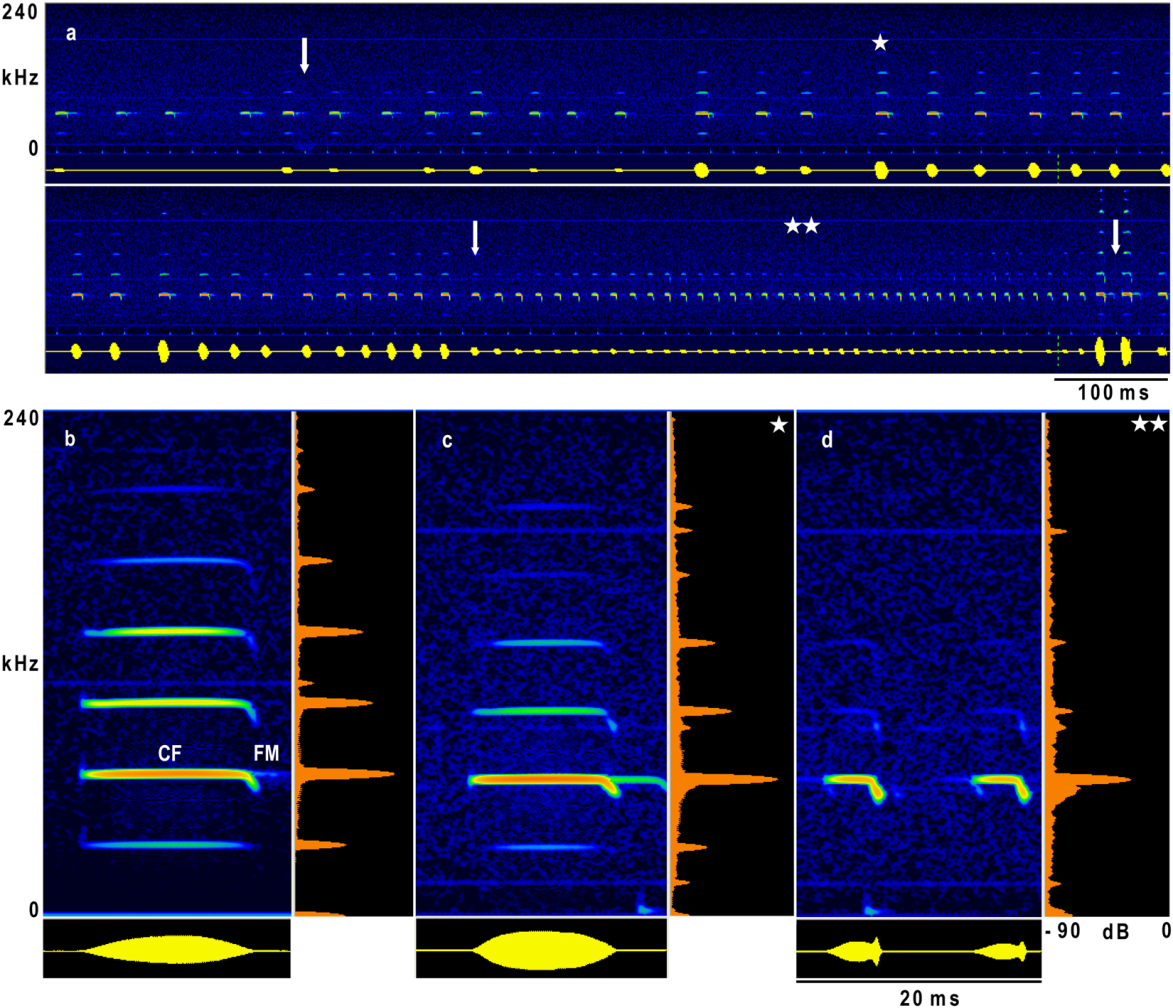
Sonogram and oscillogram (512 FFT, blackman) of an echolocation sequence in flight (**a**) with representative signals (**b**–**d**). The bat (HA 2) started to fly at the 1^st^ arrow, the 2^nd^ arrow indicates the beginning of the terminal approach, and the 3^rd^ arrow the time of landing. Sonograms, oscillograms, and averaged power spectra of representative signals of a resting signal (**b**), an echolocation signal during the orientation flight (**c**), and the terminal approach (**d**). The CF and FM component of the signal are marked in (**b**). (**c**) and (**d**) are taken from the echolocation sequence shown in (**a**) and marked with asterisks. The oscillogram in (**d**) was amplified by a factor of two.

During the course of evolution, the echolocation systems of bats have adapted to deliver information necessary to successfully perform species-specific tasks. These tasks depend on the foraging habitat, foraging strategy, and the type of prey bats feed on^1–3^. Bats of the “narrow space flutter detecting foragers” guild, comprising Rhinolophidae, Hipposideridae, and the phylogenetically distant *Pteronotus parnellii* of the family Mormoopidae, actively hunt for insects in narrow spaces where the prey echo either overlaps with or is masked by background echoes^1–5^. Flutter detecting foragers have evolved a highly specialized echolocation system to find fluttering insect prey between background targets. They emit signals which consist of a long constant frequency (CF) component followed by a shorter frequency modulated (FM) part (Fig. 1b). Hipposiderids emit shorter signals than rhinolophids and *P*. *parnellii* (reviewed in^4,6^). Signals are emitted with a high duty cycle; therefore, these bats are also called high duty cycle bats^7,8^. The CF-FM signals have the highest amplitude in the second harmonic. The frequency of the CF component of the second harmonic (CF_2_) is species-specific, but varies slightly between individuals [e.g. refs^9–12^]. In stationary bats, the CF_2_-frequency is kept almost constant and is referred to as the resting frequency (F_rest_) (reviewed in^4^)^13^.

When searching for prey, rhinolophids emit one long signal per wing beat, whereas *P*. *parnellii* often emits groups of two and hipposiderids emit groups with more signals of shorter duration^4,9,14–16^.

If a long CF-FM signal encounters a flying insect, the movement of the insect’s wings induces frequency and amplitude modulations in the CF component of the returning echoes dependent on the rhythm of the wing beat. Every time the wing is perpendicular to the impinging sound wave, an amplitude glint is produced which is up to 20 dB above the amplitude of the echo from the insect body. Simultaneously, the moving wings also produce a spectral glint, which encodes the direction of the wing movement. The generated glint pattern contains flutter information about the prey species in terms of the wing beat frequency, wing structure, wing length and size, and aspect angle^17–23^, and allows the discrimination of modulated prey echoes from unmodulated background echoes.

When flying, flutter detecting foragers lower the emission frequency to compensate for Doppler shifts (DS) of echoes from stationary targets ahead which are generated by the bat’s own movement. Doppler shift compensation (DSC) ensures that the returning echo frequency (F_echo_) is adjusted for the auditory fovea, an area on the basilar membrane of the cochlea with a highly expanded frequency representation centred around the reference frequency (F_ref_)^24–29^. F_ref_ is measured in flying bats as the average F_echo_^30^ and is always a few hundred Hertz above F_rest_. The afferent projections from the enlarged area on the basilar membrane lead to foveal areas in the brain with an overrepresentation of sharply tuned neurons with best frequencies around F_ref_ (*P*. *parnellii*^31^, *R*. *ferrumequinum*^32^, *H*. *speoris*^33^, *H*. *armiger*^34^). These neurons are very sensitive to amplitude and frequency modulations contained in the echoes from fluttering insects. Long CF-FM signals emitted at a high duty cycle, DSC, and an auditory fovea are adaptations for the detection and evaluation of echoes containing specific flutter information within unmodulated background echoes (reviewed in^4^)^35–39^.

In stationary rhinolophids and *P*. *parnellii*, the CF_2_-frequency is kept almost constant and varies only by approximately 0.1–0.2% or 200 Hz around F_rest_ within short time periods. In hipposiderids variations of up to 0.75% or 1.17 kHz have been reported^4,40^, suggesting that the CF_2_-frequency of hipposiderids may be less stable. Over periods of minutes, days, and months, distinct changes in F_rest_ have been observed in rhinolophids, hipposiderids, and *P*. *parnellii*^41–46^. In *P*. *parnellii*, F_rest_ declined by up to 120–300 Hz in experiments where bats flew freely or were swung on a pendulum, and up to 366 Hz over a 50-day period^41–43^. In rhinolophids, F_rest_ decreased gradually by 220 Hz within a three-month period, with variations of up to 2 kHz^45^. In a hipposiderid bat, maximal variations of F_rest_ of up to 4.8 kHz and 3.0 kHz on average were reported in an experiment lasting four years^44^.

DSC was studied in the laboratory in bats flying to a landing bar or when sitting on a pendulum, and in playback experiments. DSC has been described by the difference between F_ref_ and F_rest_, referred to as the offset^4,30^, and the precision with which F_echo_ is kept at F_ref_^4^. Rhinolophids and *P*. *parnellii* maintained F_echo_ with a high precision of only 0.1–0.2% around F_ref_, while in hipposiderids a much higher variation of 0.4–0.7% around F_ref_ has been reported^4^. In flight, *Rhinolophus ferrumequinum*, *Rhinolophus euryale*, and *P*. *parnellii* accurately maintain F_ref_ at approximately 150–200 Hz above F_rest_^9,13,14,16,47–51^. The hipposiderid, *Asellia tridens*, also maintains F_ref_ 150–200 Hz above F_rest_^15^, whereas for some species of the genus *Hipposideros* an offset of up to 300 to 600 Hz was reported^4^. DSC was also shown in *Hipposideros terasensis* with no further information regarding the F_ref_ or offset^52^. In a passive situation, e.g. when sitting on a pendulum or in playback experiments, rhinolophids and *P*. *parnellii* fully compensated for positive DS^40,41,49–51,53–55^ up to 8 kHz as seen in *R*. *ferrumequinum*^18,35^. However, *Hipposideros speoris* and *Hipposideros bicolor* compensated for DS only partly when sitting on a pendulum^56^, and Schuller^40^ found that *H*. *speoris* and *H*. *bicolor* did not even react to playback. These results led to the assumption that the DSC system of hipposiderids is less accurate than that of rhinolophids and *P*. *parnellii*^4,6,57^. This assumption is corroborated by behavioural audiograms and by neurophysiological data indicating that the auditory fovea seems to be less sophisticated, as it exposes less sharply tuned neurons, which results in a lower frequency selectivity^34,40,58^, (for details see^4^).

There are, however, some disadvantages in the design of studies of DSC that make it difficult to evaluate the accuracy of DSC. The echolocation behaviour of stationary bats does not necessarily reflect the behaviour of flying bats. If bats do not react to DS, it does not necessarily mean that they are not able to compensate for DS. The only approach that ensures reliable data on the precision of DSC are studies in flying bats. Further, in hipposiderids^4,40,44^, but also in *P*. *parnellii*^41–43^ and in *R*. *ferrumequinum tragatus*^46^, F_rest_ is not stable. To get reliable values of the offset, it is mandatory to measure F_rest_ directly before F_ref_ is determined. None of the studies in hipposiderids took the variations of F_rest_ into account. Furthermore, meaningful information concerning the precision of the DSC is missing. Some authors have used the ability to keep F_echo_ constant within a small frequency band just above F_rest_^18^ as a measure for precision, while others have used the offset between F_ref_ and F_rest_ as a quality measurement for DSC^41,55,56^. However, the quality of a feedback control system is indicated by the precision by which the controlled parameter is kept constant over the control range of the system^4^. In the DSC system, the precision is indicated by the accuracy by which the F_echo_ of each signal is kept at F_ref_, independent of the flight speed. Thus far, this has only been measured in *R*. *ferrumequinum* flying in a wind tunnel, where the precision of DSC was similar at all ground speeds^13^. However, none of the studies in other flutter detecting foragers have addressed this question.

For a better understanding of the precision of the DSC in a hipposiderid bat, we trained *Hipposideros armiger* to fly to a landing grid. Prior to each flight, we measured F_rest_ before the bat took off. We determined the frequency recorded at the microphone at the landing grid during flight, measured the flight speed from 3D video recordings, and calculated according to the measured speed the encountered DS and perceived F_echo_. For each flight, we precisely determined the offset between F_rest_ and the averaged F_echo_ or F_ref_ to understand their coupling, and adjusted for variations in F_rest_ and F_ref_ between flights. We also determined the precision of the DSC system by measuring whether the adjustment of F_echo_ to F_ref_ is independent of the flight speed.

## Results

### Echolocation behaviour in resting bats

**Figure 2:**
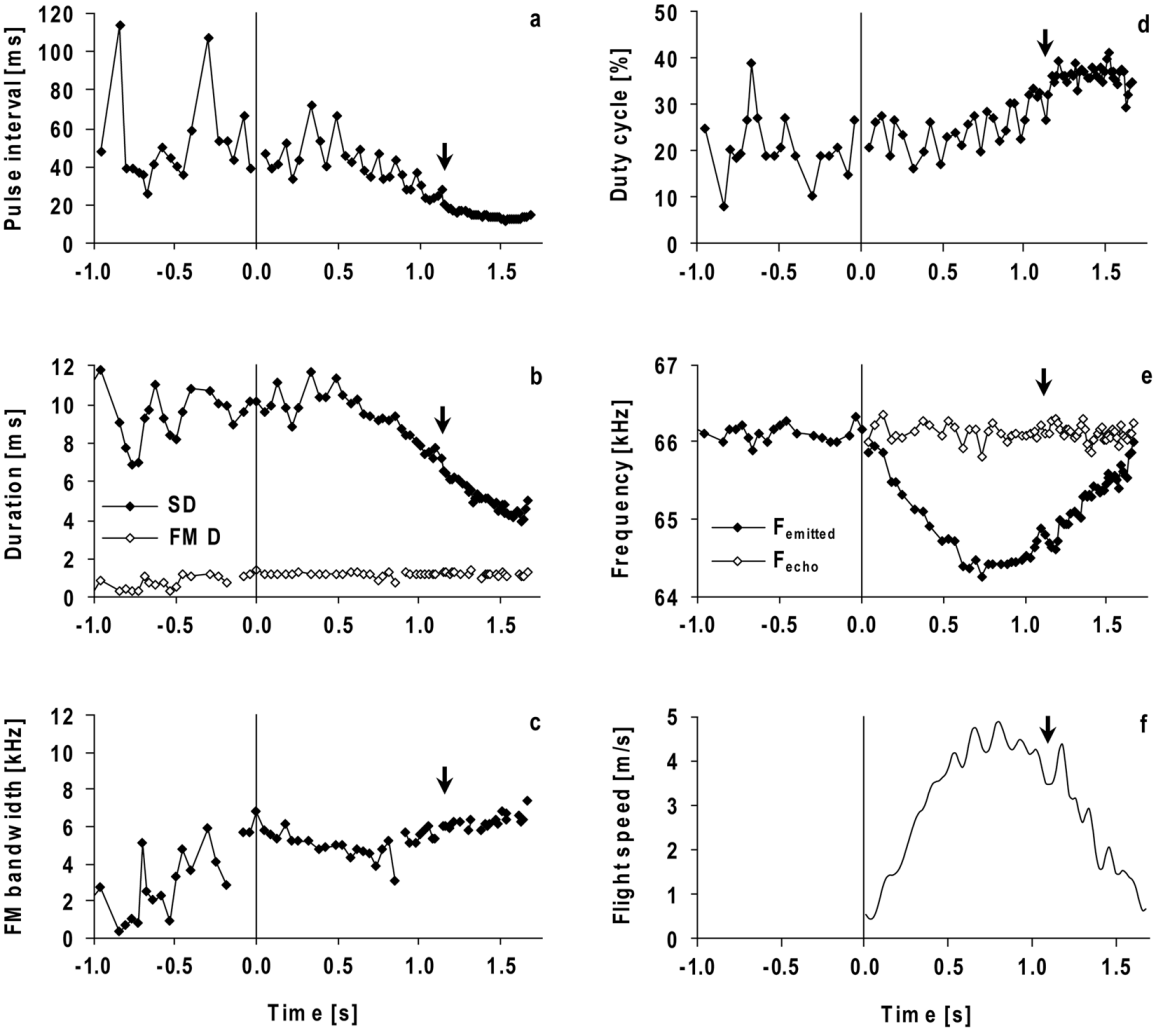
Signal parameters (**a**–**e**) and flight speed (**f**) of a representative flight of HA 2 (same sequence as shown in Fig. 1). Signal parameters (**a**–**e**) include the last 20 resting signals before take-off. The bat took off at 0 s and landed at 1.67 s. The beginning of the terminal approach is marked with an arrow. The duration of the total signal (SD) and the duration of the FM component (FM D) are shown in (**b**). Emission frequency (F_emitted_) and echo frequency (F_echo_) during flight are calculated for targets ahead (**e**) by using the flight speed (**f**). Before take-off the emission frequency corresponds to the resting frequency (F_rest_) (**e**). The averaged F_echo_ corresponds to the F_ref_.

Both *H*. *armiger* individuals (HA 1 and HA 2) exhibited similar echolocation behaviour before take-off (Table 1). They continuously emitted resting signals, generally arranged in groups, and the main energy of the multi-harmonic signals was concentrated at the second harmonic (Fig. 1b). With a mean duration of 9.7 ms the calls of HA 1 were 0.7 ms shorter than the calls of HA 2 (t (38) = 2.13, p = 0.040) (Table 1). For HA 1, the FM component was longer in duration and higher in bandwidth (t (38, 38) ≥ 5.34, p < 0.0001) than HA 2. Hence, 88% (HA 1) and 91% (HA 2) of the total signal duration was determined by the CF component. The pulse interval and duty cycle were highly variable before the bats started to fly (Table 1, Fig. 2a,d). Both bats had an average pulse interval of around 70 ms (t (38) = 0.51, p = 0.61). The duty cycle was 16% in HA 1 and 18% in HA 2 (t (38) = 3.35, p = 0.0019). F_rest_ was not stable (see below).

**Table 1:** Mean ± SD of the signal parameters of resting signals of *H*. *armiger* before take-off [bat 1 (HA 1) and bat 2 (HA 2), n = 400 per bat].

	**HA 1**	**HA 2**
Pulse interval [ms]	73.5 ± 39.3	70.4 ± 45.7
Signal duration [ms]	9.7 ± 1.5	10.4 ± 1.8
Duty cycle [%]	15.5 ± 5.3	18.3 ± 7.1
FM duration [ms]	1.2 ± 0.6	0.9 ± 0.4
FM bandwidth [kHz]	6.5 ± 2.1	3.3 ± 1.9

### Echolocation behaviour in flying bats

*H*. *armiger* flew stereotyped flight paths straight to the landing grid [Supplementary Fig. S1]. Before landing on the grid, they turned upside down. After take-off, the bats emitted search or orientation signals, which were often arranged in groups (Fig. 1a,c). About 1 s before landing the bats switched to the initial approach, indicated by an increase of pulses per group and a reduction of the pulse interval and signal duration. At 1.6 ± 0.3 m (HA 1) and 1.7 ± 03 m (HA 2) or 640 ± 90 ms (HA 1) and 660 ± 70 ms (HA 2) before landing on the grid, the bats started the terminal approach and emitted a long terminal group with 41 ± 4 (HA 1) and 44 ± 4 (HA 2) short signals on average (Fig. 1a,d). The start of the terminal approach and the number of signals did not differ between the bats (t (18, 18) ≤ 1.59, p ≥ 0.1296). In flight, the bats emitted multi-harmonic signals with the main amplitude in the second harmonic, as they did before take-off. However, the other harmonics were more strongly suppressed than in resting signals (Fig. 1b,c). Harmonic suppression was even more distinct in the terminal group (Fig. 1d). The CF component was maintained up to the last signal of the terminal group, and sometimes the FM component had a higher peak amplitude than the CF component (Fig. 1a,d). In search or orientation signals, the averaged pulse interval and signal duration differed between bats. HA 1 had a mean pulse interval of 38 ± 7 ms and HA 2 of 48 ± 12 ms; the mean call duration of 9.6 ± 0.8 ms was also shorter in HA 1 than in HA 2 who emitted signals with a duration of 10.5 ± 1 ms (t (18, 18) ≥ 3.95, p ≤ 0.0009). During approach, both parameters decreased continuously the closer the bat was to the landing grid. In the terminal group, the pulse interval declined to a minimum of 12.1 ms (HA 1) and 11.4 ms (HA 2), and signal duration to a minimum of 4.3 ms (HA 1) and 3.2 ms (HA 2) (Fig. 2a,b). When the bats started to fly, the duty cycle of 26 ± 4% in HA 1 was slightly higher than the duty cycle of 23 ± 4% in HA 2 (t (18) = 7.31, p < 0.0001) and increased during approach up to 41% in HA 1 and 48% in HA 2 (Fig. 2d). HA 1 reduced the duration of the FM component from 1.4 ± 0.2 ms during search flight to 1.3 ± 0.2 ms in the terminal approach (t (18) = 3.8, p = 0.0013), whereas HA 2 did not change the duration of the FM component significantly [FM duration, 1.25 ± 0.1 ms during search flight, 1.23 ± 0.1 ms in the terminal approach (t (18) = 0.94, p = 0.3588)]. In search flight, the bandwidth of the FM component in HA 1 (7.5 kHz) was much higher than in HA 2 (5.2 kHz) (Z = −3.7, n = 10, p = 0.0002). Both bats exhibited an increase in bandwidth from search to terminal approach, in HA 1, who had the higher bandwidth in search flight, by 290 Hz on average and in HA 2 by 940 Hz (t (18) = 5.37, p < 0.0001) (Fig. 2b). Overall, the sweep rate of the FM was increased in the terminal FM, either by a reduction in duration and increase of bandwidth (HA 1), or an increase in bandwidth alone (HA 2, Fig. 2c).

### Doppler shift compensation

**Figure 3:**
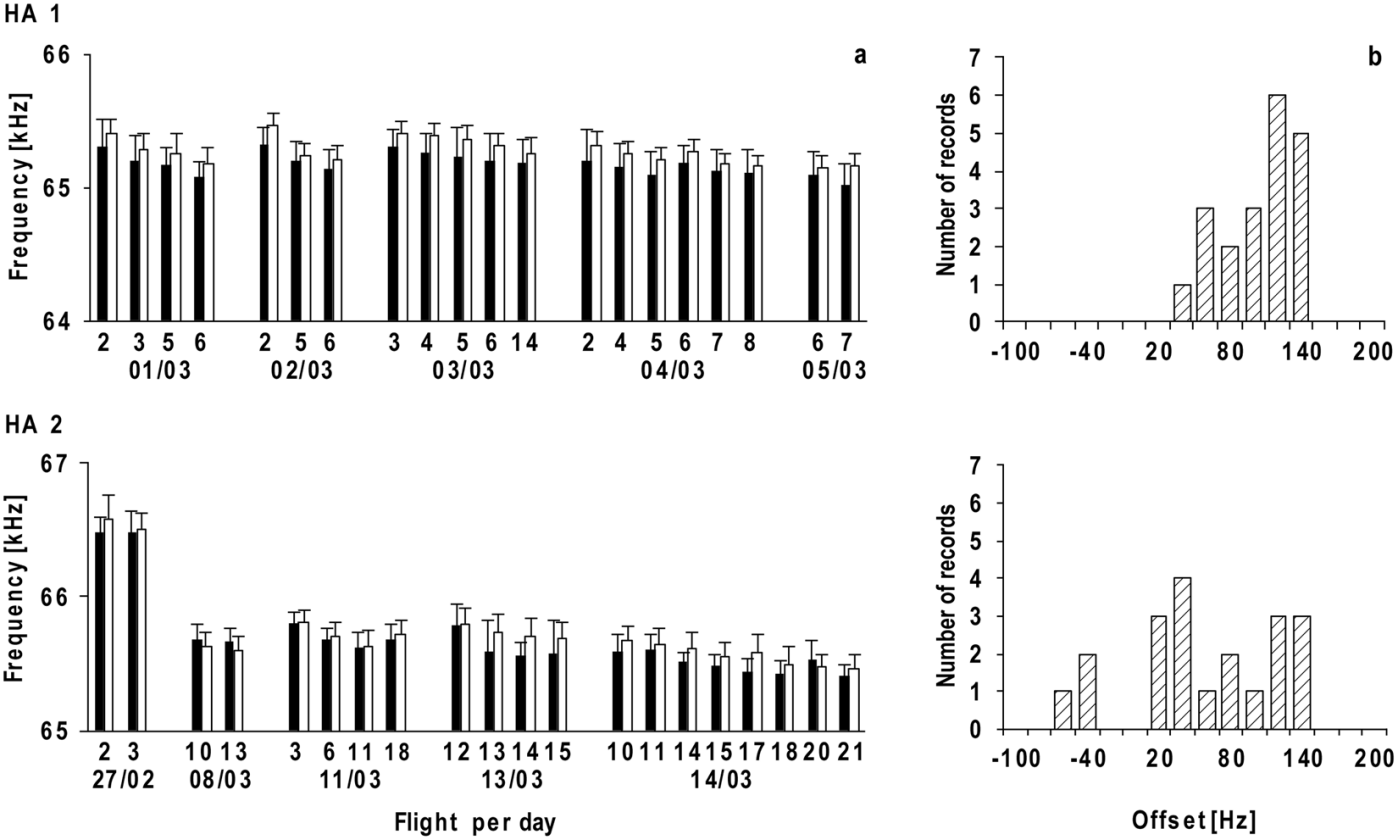
Reference frequency (F_ref_) and corresponding resting frequency (F_rest_). Mean ± SD of the CF_2_- or resting frequencies before the bats take-off (black bars) and F_ref_ (white bars) for 20 flights per bat (**a**). The x-axis shows the number of the flight within one session. The distribution of the offset between F_rest_ and F_ref_ is shown in (**b**).

**Figure 4:**
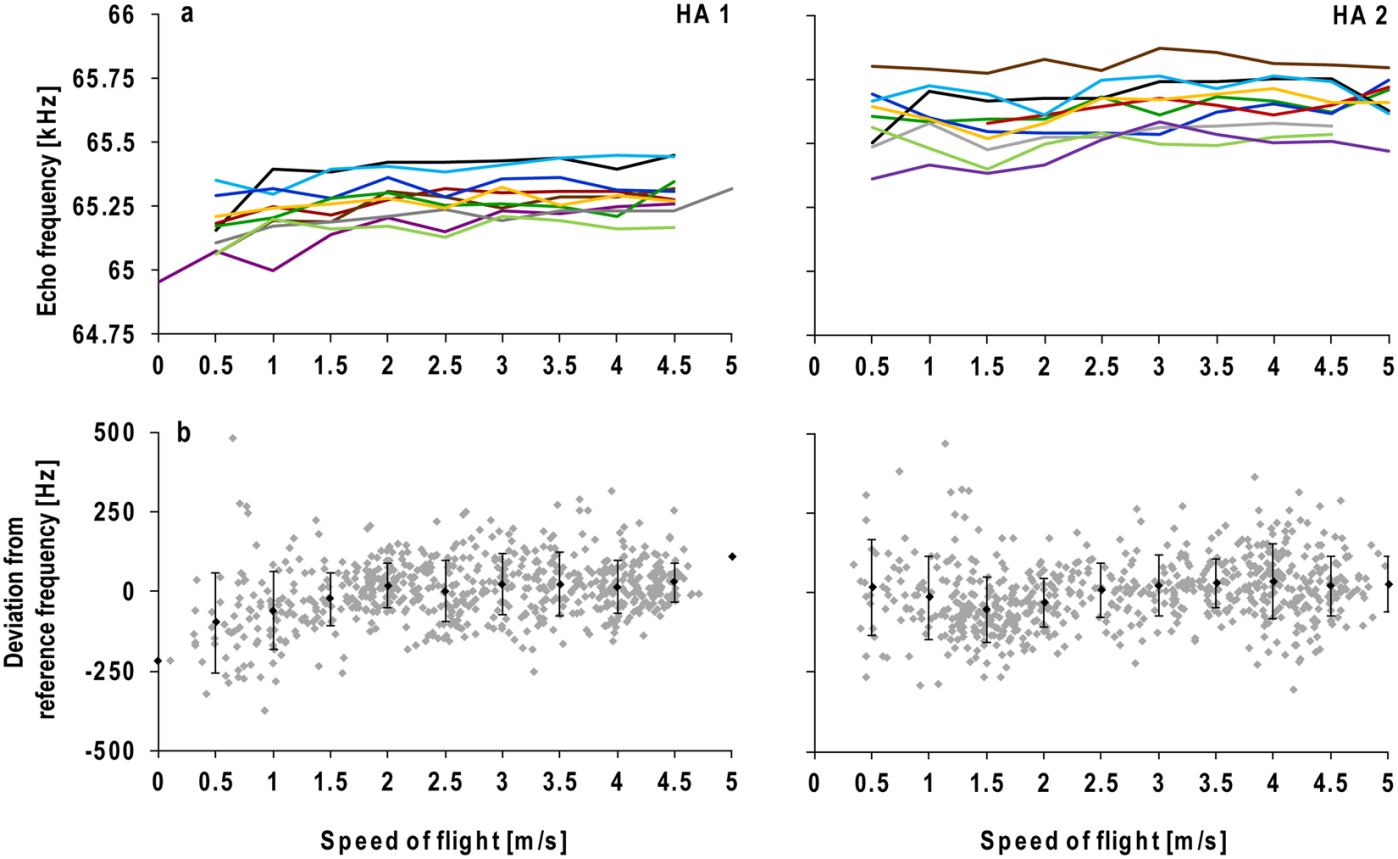
Echo frequency plotted against flight speed for HA 1 and HA 2. Means of the echo frequencies (F_echo_) calculated for 0.5 m/s classes of ten flights (coloured) (**a**). Deviation of F_echo_ from the reference frequency of the corresponding flight calculated for each call of ten flights (grey dots). Black dots indicate means (±SD) calculated for 0.5 m/s classes (**b**).

**Figure 5:**
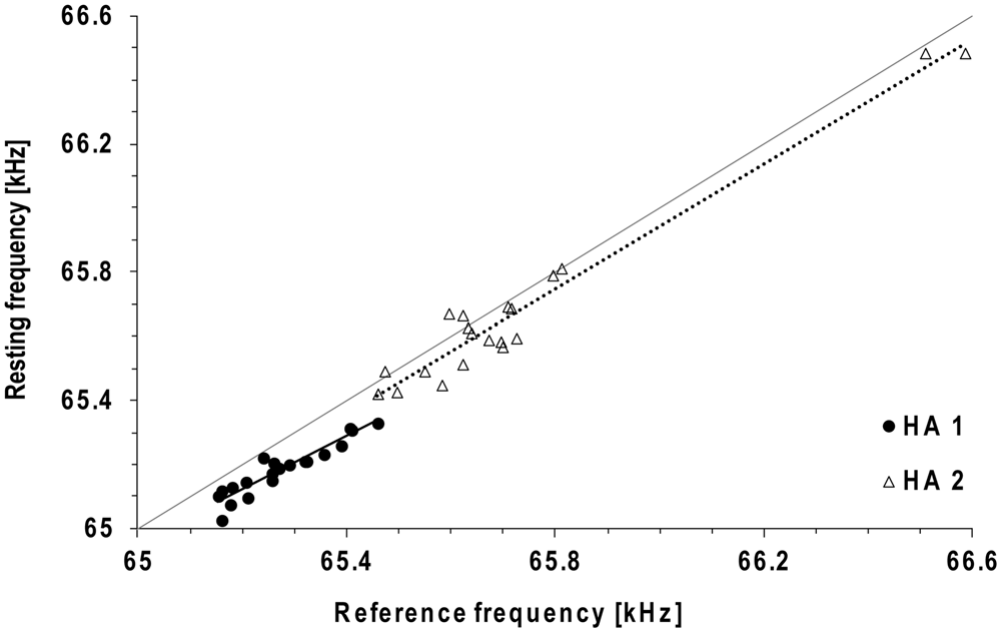
Correlation between the means of resting frequency and reference frequency. For each bat, 20 flights with linear regression lines are shown. The grey line indicates the angle bisector.

In flight, *H*. *armiger* lowered the frequency of the CF component of the CF-FM signals, such that the frequency of the CF_2_ component of the echoes from ahead (F_echo_) was kept almost constant around F_ref_, which was determined as the average of all calculated echo frequencies of the corresponding flight (Fig. 2e). The lowest emission frequency was measured when flight speed reached a maximum of 4.7 m/s (Fig. 2e,f). In single flights, standard deviations ranging from 80 Hz to 170 Hz were measured. The average standard deviation for all flights of both bats was 110 Hz (Fig. 3a), corresponding to a deviation of only 0.17% from F_ref_. The averaged standard deviation of 140 Hz around F_rest_ was slightly higher, corresponding to 0.21%. F_rest_ and F_ref_ were not stable; they differed from flight to flight and from day to day (Figs 3a and 4a). However, F_ref_ and F_rest_ varied in a systematic way during flights. The correlation of F_rest_ with F_ref_ was highly significant (HA 1: Spearman r = 0.92, p < 0.0001; HA 2: Spearman r = 0.84, p < 0.0001) and the regression lines were almost parallel to the bisector, indicating that F_rest_ and F_ref_ were tightly coupled (Fig. 5). In nine out of ten sessions, F_ref_ and F_rest_ decreased with the number of flights performed by the bats but started at approximately the same initial level on the next day. F_ref_ declined by 130 Hz on average between the first and last flight within a session (Fig. 3a). The highest difference between the first and last flight within one session was 250 Hz (HA 1, 02/03, Fig. 3a). Furthermore, in HA 2 we observed a decline of F_ref_ by 1 kHz over the course of just nine days (27/02 and 08/03, Fig. 3a).

Since F_ref_ and F_rest_ were not stable, we calculated the offset between F_rest_ and F_ref_ for every single flight. The offset between F_rest_ emitted just before the bat took off and the calculated F_ref_ during flight measured between 5 and 140 Hz, except for 3 out of 20 flights in HA 2 in which F_rest_ was higher than the calculated F_ref_. The mean difference between F_rest_ and F_ref_ was 90 Hz in HA 1 and 70 Hz in HA 2 (excluding the three negative values), which corresponds to an offset of approximately 0.12% (Fig. 3b).

The precision with which F_echo_ is kept at F_ref_, independent of the encountered DS and thus independent of flight speed, indicates the quality of the DSC feedback control system. The correlation between the deviation of F_echo_ from F_ref_ and the flight speed, as a measure of precision, was significant in both bats; however, F_echo_ was not predicted by flight speed as indicated by the coefficient of determination, which was smaller than 0.1 (HA 1: r² (680) = 0.080, p < 0.0001; HA 2: r² (670) = 0.056, p < 0.0001). Variation was higher at low flight speeds, indicated by higher standard deviations (Fig. 4b).

## Discussion

Rhinolophids, hipposiderids, and the mormoopid bat, *P*. *parnellii*, use the flutter detecting echolocation strategy to find prey. The echolocation systems of flutter detecting foragers are described by F_rest_ and F_ref_. F_rest_ is determined by the average frequency of the CF component of the CF-FM signals in stationary bats, and F_ref_ is measured in bats performing DSC as the average frequency of the CF component in the echoes returning from ahead. In rhinolophids and *P*. *parnellii*, F_rest_ and F_ref_ are almost constant within short time periods; offsets between F_rest_ and F_ref_ of only 150–200 Hz^9,14,30,48,50,51^ have been measured. However, it has been reported that in hipposiderids F_rest_ was less stable^40^, F_echo_ was regulated with less precision around F_ref_, (reviewed in^4^), and offsets of 300 to 600 Hz were relatively high. This led to the assumption that the DSC feedback system of hipposiderids may be less precise than in rhinolophids and *P*. *parnellii*^40,56^.

The variability of F_rest_ and F_ref_ in hipposiderids poses the problem that the values given for F_rest_, F_ref_, and the offset between them strongly depend on the instant in time when they are measured. To overcome this problem, we adjusted for frequency variations by measuring F_ref_ for every flight and F_rest_ just prior to take-off, and used these values to calculate the offset. Both F_rest_ and F_ref_ were rather stable when measured for every single flight, with deviations similar to those observed in rhinolophids and *P*. *parnellii*. However, F_rest_ and F_ref_ varied up to 230 Hz and 250 Hz, respectively, within a session and between daily sessions. In one bat, a drop of 1 kHz over 10 days was observed.

Although we found a high variability in F_rest_ and F_ref_, there was always a tight coupling between the two frequencies. F_rest_ was slightly below F_ref_, and both F_ref_ and F_rest_ changed in the same manner. The tight coupling accounts for a rather small mean offset of 80 Hz measured in single flights, independent of the changes in absolute frequency, such that either frequency can be used to predict the other. The offset of 80 Hz measured in *H*. *armiger* is within the range of 150–200 Hz reported for rhinolophids and *P*. *parnellii*^9,14,40,48,50,51^. This suggests a similar coupling mechanism for all DSC bats. The variations of F_ref_ and F_rest_ in hipposiderids were not considered in previous studies and most likely account for the high deviations of F_ref_, F_rest_, and offset reported in this family. Only Gustafson and Schnitzler^15^ measured the offset in a similar way in *A*. *tridens*, which explains why their results were in the same range of values as found in this study.

Stationary flutter detecting foragers emit signals with an F_rest_ always slightly below F_ref_. This has the advantage that echoes from objects moving towards the bat will generate positive DS and are thus higher in frequency than the emitted signals. These echo frequencies will fall into the range of F_ref_ where the auditory system is most sensitive. F_rest_ being below F_ref_ may have the function of an alarm system for prey or predators moving towards the bat. The tight coupling of F_rest_ to F_ref_ ensures that this system also functions with a variable F_ref_.

The auditory fovea of flutter detecting foragers consists of an enlarged area on the basilar membrane with an overrepresentation of frequencies around F_ref_^24,29^ that projects into higher auditory centres in the brain (e.g. in hipposiderids:^33,34,59^) with sharply tuned neurons specialized for the evaluation of flutter information. DSC bats lower the emission frequency to keep the echo frequency constant at F_ref_. High precision DSC ensures an optimal flutter evaluation. A variable F_ref_ implies that the frequency that leads to maximal activation of the auditory fovea changes somewhat for reasons that are yet unknown. Liu *et al*.^46^ came to a similar conclusion when explaining the variation of F_rest_ in *R*. *ferrumequinum tragatus*. They housed pairs of bats and recorded their echolocation and social calls. While social calls did not change in frequency, they found day-to-day changes in F_rest_ of up to 900 Hz. They concluded that changes in F_rest_ mirror changes in the tuning of the auditory fovea. We assume that the resonance properties of the basilar membrane in the cochlea change, influencing the maxima of the travelling wave and with it the position at which a specific frequency is represented. This is supported by studies in *P*. *parnellii* where body temperature and/or flight activity influenced the cochlear resonance frequency^42,60^, and also F_rest_ and F_ref_^43^. F_rest_ and F_ref_ were positively correlated with body temperature, exhibiting changes of 93 Hz/°C and 90 Hz/°C, respectively. Huffman and Henson^43^ suggested that the cochlear resonance frequency is affected by changes in body temperature, inducing shifts in F_rest_ and F_ref_. We assume that in our flight experiments the foveal resonance properties in the cochlea of *H*. *armiger* changed, possibly due to variations in body temperature, and that these changes are mirrored by the shift of F_rest_ and F_ref_. DSC bats thus adjust F_echo_ or F_ref_ such that the foveal resonance area on the cochlea and their projections into the auditory brain are maximally activated^4^.

In feedback control systems the best measure to assess quality is the precision with which the parameter under control (i.e. F_echo_) is kept at the reference parameter (i.e. F_ref_)^4^. In a first approximation the precision can be estimated by the deviation of F_echo_ from F_ref_. In rhinolophids and *P*. *parnellii* this deviation was measured as the width of the frequency range of F_echo_ around F_ref_ and reached values of 0.1–0.2%. In hipposiderids the estimated values were much higher at 0.4–0.7%^4^, except for *A*. *tridens* in which the deviation was distinctly smaller^15^. In *H*. *armiger*, we measured an averaged standard deviation of 110 Hz, corresponding to a deviation of only 0.17% from F_ref_. These values are similar to the variability measured in rhinolophids and *P*. *parnellii*.

The use of the averaged standard deviation as an indicator of precision is only appropriate if bats are able to maintain F_echo_ at F_ref_ independent of the flight speed. We controlled for this assumption by calculating the deviation of F_echo_ from F_ref_ for every single echo and related it to the flight speed (Fig. 4). Although the correlation between the deviation of F_echo_ from F_ref_ and flight speed was significant, biased by the large sample size, a coefficient of determination of 0.080 or lower indicates a poor model fit with less than 10% of the F_echo_ variance explained by the influence of the flight velocity. This implies that the F_echo_ is rather independent from flight speed. The higher variation at low flight speeds, indicated by the higher standard deviations, is due to the accelerated flight during take-off and landing. Fast changes reduce the precision of the slow feedback control system^61^. In flying *R*. *ferrumequinum*, the performance of DSC at different flight speeds was measured in a wind tunnel^13^. Independent of the ground speed, the bats adjusted F_echo_ in such a way that the offset to F_rest_ stayed the same and was similar to the offset measured in flying bats under normal conditions. To describe the precision of DSC systems, we suggest testing whether a species compensates for DS independent of flight speed and, if yes, to determine the average standard deviation of F_echo_ relative to that of F_ref_.

In playback experiments with stationary bats that simulate different flight speeds by presenting echoes with positive DS up to 8 kHz, *R*. *ferrumequinum* fully compensated for DS and kept F_echo_ at F_ref_ with standard deviations of 30 to 40 Hz^30,35^. Comparable playback data for *P*. *parnellii* are missing; however, similar DSC performance in flight suggests a similar precision^14,51^. In contrast, hipposiderids failed to react in playback experiments^40^. This does not necessarily prove that the feedback control system of hipposiderids is less precise, as these experiments probably were not sufficiently naturalistic for the bats. In conclusion, our data from *H*. *armiger* and data from *A*. *tridens*^15^ suggest that most likely all hipposiderids compensate for DS with the same precision as rhinolophids and *P*. *parnellii*.

When flying towards the landing grid, *H*. *armiger* already emitted groups of signals during orientation or search flight. The beginning of the approach phase is therefore not as obvious as in other bats. However, a reduction of signal duration and pulse interval, and an increase in duty cycle and number of signals per group indicated that the approach behaviour started at about 1 s before landing. The terminal group had the highest number of signals, 43 on average. The echolocation behaviour of *H*. *armiger* in our landing task was similar to the behaviour of other hipposiderids in a comparable behavioural situation^15,52^. There are, however, distinct differences to the approach behaviour of rhinolophids. In orientation or search flight rhinolophids often emit one long signal per wing beat, whereas hipposiderids emit several shorter signals per wing beat. In the initial approach, the grouping of signals was less regular than in rhinolophids and the terminal group comprised a much higher number of signals than the terminal group of other flutter detecting foragers^9,14–16^. In general, hipposiderids operate with the shortest signals of all flutter detecting foragers. Nevertheless, due to the high repetition rate, they have a high duty cycle.

## Conclusion

In *H*. *armiger*, the precision of DSC, measured as the deviation of F_echo_ from F_ref_ during flight, was similar to that in horseshoe bats and *P*. *parnellii*, and the precision of DSC was independent of the flight speed. F_ref_ and F_rest_ varied from flight to flight, but F_ref_ and F_rest_ were tightly coupled. The offset between the two frequencies was similar to the offset measured in rhinolophids and *P*. *parnellii*. The variation of F_ref,_ and with it, F_rest_, may indicate that the resonance frequency of the foveal area in the cochlea varies and that the bats adjust F_echo_ or F_ref_ such that the foveal resonance area on the cochlea and its projections into the auditory brain are maximally activated to ensure an optimal evaluation of flutter information. This implies that the DSC system of hipposiderids is of similar precision to that of horseshoe bats and *P*. *parnellii*. The frequency variations may explain why in former studies it has been assumed that DSC in hipposiderid bats is incomplete.

There are, however, differences in the flutter evaluation systems between families that might affect the quality of flutter information extracted from echoes^4^. Rhinolophids and *P*. *parnellii* emit long signals and increase their duty cycle by producing longer signals as a reaction to flutter information. The echo of a single signal comprises the glints of several wing beats of an insect. Signals of hipposiderids are much shorter and the duty cycle is increased by emitting more signals; thus flutter information is encoded over several echoes (reviewed in^4,6^). Additionally, the auditory fovea of rhinolophids and *P*. *parnellii* is largely expanded around F_ref_^24,28^ whereas the auditory fovea of hipposiderids is less developed (40, 58). Further, the neurons in foveal areas of the auditory pathway are more sharply tuned in rhinolophids and *P*. *parnellii* than in hipposiderids, indicated by higher Q_10dB_ values. Thus, hipposiderids may be less able to decode detailed flutter information compared with rhinolophids or *P*. *parnellii*. The precise DSC, however, enables them to keep F_echo_ exactly at F_ref_ to distinguish moving prey echoes from non-moving background echoes.

## Materials and Methods

### Animals

We conducted the experiments with two adult Great Leaf-nosed Bats [*Hipposideros armiger* (Hodgson, 1835)], captured in the Ba Be National Park in northern Vietnam (Permission No. 129/STTNSV from May 13^th^, 2009, granted to the Vietnamese Institute of Ecology and Biological Resources, Hanoi). They were housed in two aviaries (3.2 × 1.25 × 2 m and 2.4 × 1.2 × 2 m) but had access to a room (6.0 × 3.6 × 3 m) where they could freely fly together. Abiotic conditions were kept constant (12:12 hours light/dark cycle, 26.6 ± 2 °C and 60 ± 5% humidity) and bats had free access to water and mealworms (*Tenebrio molitor*). Food was supplemented with vitamins (Nutrical®), fatty acids (Efaderm®), and minerals (Korvimin®). Additionally, they were hand-fed with large insects, including crickets (*Gryllus* spp. and *Acheta domestica*), grasshoppers (*Schistocerca gregaria*), and beetles (*Zophobas morio*, *Pachnoda marginata*).

### Ethical statement

The animal facility of the Institute for Neurobiology at the University of Tübingen in which the bats were kept is approved by the Regierungspräsidium Tübingen (AZ: 35/9185.46/Uni Tü) according to §11 of the German Animal Welfare Law. We and the animal welfare officers of the Faculty of Science and of the University of Tübingen agreed that our experiments did not require animal experimentation approval, as it could be excluded that the bats were exposed to procedures that cause pain, distress, suffering, or harm according to Directive 2010/63/EU and §7 of the German Animal Welfare Law. Bats were accustomed to being handled and familiar with the flight room. They always had access to water and food and could fly without restriction in the flight room. Using large insects favoured by the bats as positive reinforcement, they learned to fly from a starting bar to their preferred landing site in the room. For similar experiments, the approval authority agreed that a permit is not necessary (by letter from the Regierungspräsidium Tübingen, March 29^th^, 2012).

### Experimental setup

All experiments were carried out in complete darkness in a 6.0 × 3.6 × 3 m flight room. The walls and the ground were covered with sound absorbent foam to reduce echoes. Bats were trained by positive reinforcement with favoured insects to fly freely from a starting bar (1.2 m above ground) to a landing grid positioned at the preferred landing site of each bat. The grid of bat 1 (HA 1) was positioned 2.7 m above ground at a distance of 3.5 m from the starting bar. Bat 2 (HA 2) landed on a grid fixed to the wall, 1.4 m above the ground, at a distance of 4.3 m from the starting bar. The ultrasonic microphone was positioned 3 cm above each landing grid (Supplementary Fig. S1). All other recording devices were behind the starting bar and did not affect the bats’ behaviour. Except for the walls of the room, there were no objects along the flight paths.

### Data recordings and analysis

The flight behaviour of the bats was recorded with two IR cameras (Sanyo IR CCD, Japan) at a rate of 50 Hz. Each half frame was illuminated for 1 ms by two infrared strobe flashes. The videos were stored on Panasonic DVC mini-tapes using two camcorders (Sony, DCR-TR V50E, Japan). After digitizing, the recordings were analysed (SIMI Motion Reality Motion Systems, 7.5.293) to reconstruct the 3D flight path (mean reconstruction error of 3.2 cm) and to calculate the flight speed.

Sound recordings were made with PC-Tape (Animal Physiology, University of Tübingen). The echolocation signals were picked up through a custom-made ultrasonic microphone (nearly flat frequency response, at 100 kHz 4 dB less sensitive than at 20 kHz), digitized (480 kHz, 16 bit), and stored as wav-files. Sound recordings were synchronized with the video recordings. For analysis, the recordings were displayed as colour sonograms (FFT 512, Blackman window, dynamic range 90 dB) in a window of 512 × 512 pixels with a frequency range of 35–95 kHz and a duration of 50 ms (custom-written software Selena, Animal Physiology, University of Tübingen). Due to autopadding and interpolation in time, we achieved a resolution of Δf = 110 Hz and Δt = 0.1 ms. Sound parameters were calculated using a MATLAB routine (Matlab®, 7.7.0.402, 2009b, written by Peter Stilz). The beginning and end of an echolocation signal was defined at −30 dB below best amplitude, and the beginning of the terminal FM component at 660 Hz below the CF_2_-frequency corresponding to approximately 1% of the CF_2_-frequency.

To determine the precise CF_2_-frequency recorded at the microphone (F_M_) to calculate the DSC, we displayed the signals between 63 and 69 kHz using an FFT with 8192 points (zero padding), resulting in a frequency resolution of 11.5 Hz. The CF_2_-frequency was measured at the peak amplitude of the CF component.

To calculate the emitted CF_2_- or signal frequency (F_S_) and the F_echo_ from the frequency recorded by the microphone, we used the equations published by Schnitzler^13^. The signal frequency is given by equation (1)

1 $${{\rm{F}}}_{{\rm{S}}}={{\rm{F}}}_{{\rm{M}}}\times ({\rm{c}}-{{\rm{v}}}_{{\rm{B}}})/{\rm{c}}$$

where F_M_ is the recorded frequency at the microphone, v_B_ the speed of the bat, and c the velocity of sound (343 m/s).

In situations where the bat was not flying directly towards the microphone, e.g. immediately after take-off, the Doppler shift at the microphone was smaller than the calculated Doppler shift from ahead according to the cosine of the angle between the flight and microphone direction. Therefore, the calculated signal frequency (F_S_) was slightly too high. At low flight speeds and the small angles after take-off these deviations were very small, e.g. at an angle of 30° and a flight speed of 1 m/s the deviation was only 28 Hz. The deviations reduced to 0 Hz when the bats changed flight direction and flew directly towards the recording microphone. Therefore, these deviations were not considered.

To determine the frequency of the echo (F_echo_) which the bat receives from stationary targets directly ahead, two Doppler shifts have to be added^13^. If v_B_ is small in comparison to c, equation (2) gives a good approximation for F_echo_

2 $${{\rm{F}}}_{{\rm{echo}}}={{\rm{F}}}_{{\rm{S}}}+{{\rm{F}}}_{{\rm{S}}}\times {{\rm{2v}}}_{{\rm{B}}}/{\rm{c}}.$$

In total, we analysed the flight and echolocation behaviour of 20 flights for each bat recorded over a time course of 16 days. We first determined the resting frequency (F_rest_) by measuring the averaged CF_2_-frequency of the last 20 echolocation signals while the bat was still hanging at the starting bar before take-off. For every single flight, we determined the reference frequency (F_ref_) as the mean of all calculated F_echo_. Further, we calculated the standard deviation with which F_echo_ varied around F_ref_. Since data were not normally distributed (Shapiro Wilk test, p < 0.05), we evaluated the Spearman correlation between the mean of F_rest_ and the F_ref_ for all 20 flights per bat.

An indicator of the quality of the DSC feedback control system is the precision with which F_echo_ is kept at F_ref_ independent of the flight speed. Since F_echo_ varied between flights, we subtracted the calculated F_echo_ of every single signal from the F_ref_ (which is the mean of the calculated echo frequencies of the whole sequence) in 10 flights per bat, plotted it against flight speed, and tested whether the deviation depends on flight speed with a Pearson correlation. Further, we calculated the means of this deviation from F_ref_ within flight speed classes of 0.5 m/s. In 10 of the 20 flights per bat, we analysed the echolocation behaviour and measured pulse interval, signal duration, duty cycle, bandwidth, and duration of the FM component. We visually checked for a normal distribution using histograms and normal quantile plots. We tested whether the echolocation behaviour differed between individuals using a t-test or Wilcoxon rank sum test.

### Data availability

The datasets generated and analysed during the current study are available from the corresponding author on reasonable request.

## Electronic supplementary material

Supplementary information

## References

[1] Schnitzler H-U, Kalko EKV (2001). Echolocation by insect-eating bats. BioScience.

[2] Schnitzler H-U, Moss CF, Denzinger A (2003). From spatial orientation to food acquisition in echolocating bats. Trends Ecol. Evol..

[3] Denzinger A, Schnitzler H-U (2013). Bat guilds, a concept to classify the highly diverse foraging and echolocation behaviors of microchiropteran bats. Front. Physiol..

[4] Schnitzler H-U, Denzinger A (2011). Auditory fovea and Doppler shift compensation: adaptations for flutter detection in echolocating bats using CF-FM signals. J. Comp. Physiol. A.

[5] Denzinger, A., Tschapka, M. & Schnitzler, H.-U. The role of echolocation strategies for niche differentiation in bats. *Can J Zool*, doi:10.1139/cjz-2017–0161 (2017).

[6] Fenton MB, Paul AF, Ratcliffe JM (2012). Evolution of high duty cycle echolocation in bats. J. Exp. Biol..

[7] Neuweiler, G. & Fenton, M. B. Behaviour and foraging ecology of echolocating bats in *Animal* Sonar (eds Nachtigall, P. E. & Moore, P. W. B.) 535–549 (New York: Plenum Press, 1988).

[8] Fenton, M. B. Natural history and biosonar signals in *Hearing by bats* (eds. Popper, A.N. & Fay, R.R.) 37–86 (New York: Springer, 1995).

[9] Schnitzler H-UDU-O (1968). der Hufeisen-Fledermäuse (Chiroptera-Rhinolophidae) in verschiedenen Ortungssituationen. Z. Vergl. Physiol..

[10] Heller K-G, Helversen O (1989). Resources partitioning of sonar frequency bands in rhinolophoid bats. Oecologia.

[11] Francis, C. M. & Habersetzer, J. Interspecific and intraspecific variation in echolocation call frequency and morphology of horseshoe bats, *Rhinolophus* and *Hipposideros* in *Bat Biology and Conservation* (eds Kunz, T. H. & Racey, P. A.). 169–179 (Washington and London: Smithsonian Institution Press, 1998).

[12] Siemers BM, Beedholm K, Dietz C, Dietz I, Ivanova T (2005). Is species identity, sex, age or individual quality conveyed by echolocation call frequency in European horseshoe bats?. Acta Chiropt..

[13] Schnitzler H-U (1973). Control of Doppler shift compensation in the Greater Horseshoe bat. Rhinolophus ferrumequinum. J. Comp. Physiol..

[14] Schnitzler H-U (1970). Die Echoortung bei der Fledermaus *Chilonycteris rubiginos*a. Z. Vergl. Physiol..

[15] Gustafson Y, Schnitzler H-U (1979). Echolocation and obstacle avoidance in the hipposiderid bat *Asellia tridens*. J. Comp. Physiol. A.

[16] Tian B, Schnitzler H-U (1997). Echolocation signals of the Greater Horseshoe bat (*Rhinolophus ferrumequinum*) in transfer flight and during landing. J. Acoust. Soc. Am..

[17] Goldman LJ, Henson OW (1977). Prey recognition and selection by the constant frequency bat, *Pteronotus p*. *parnellii*. Behav. Ecol. Sociobiol..

[18] Schnitzler, H.-U. & Henson, Jr. O. W. Performance of airborne animal sonar systems: I. Microchiroptera in *Animal* Sonar Systems (eds Busnel, R. G., & Fish, J. F.) 109–181 (US: Springer, 1980).

[19] Schnitzler, H.-U., Menne, D., Kober, R. & Heblich, K. The acoustical image of fluttering insects in echolocating bats in *Neuroethology and behavioral physiology* (eds Huber, F. & Markl, H.) 235–250 (Berlin: Springer, 1983).

[20] Schuller G (1984). Natural ultrasonic echoes from wing beating insects are encoded by collicular neurons in the CF-FM bat. Rhinolophus ferrumequinum. J. Comp. Physiol. A.

[21] Von der. Emde G, Menne D (1989). Discrimination of insect wingbeat-frequencies by the bat *Rhinolophus ferrumequinum*. J. Comp. Physiol. A.

[22] Kober R, Schnitzler H-U (1990). Information in sonar echoes of fluttering insects available for echolocating bats. J. Acoust. Soc. Am..

[23] Von der. Emde G, Schnitzler H-U (1990). Classification of insects by echolocating greater horseshoe bats. J. Comp. Physiol. A.

[24] Bruns V (1976). Peripheral auditory tuning for fine frequency analysis by the CF-FM bat, *Rhinolophus ferrumequinu*m. II. Frequency mapping in the cochlea. J. Comp. Physiol..

[25] Henson MM (1978). The basilar membrane of the bat. Pteronotus p. parnellii. Am. J. Anat..

[26] Schuller G, Pollak G (1979). Disproportionate frequency representation in the inferior colliculus of Doppler-compensating Greater Horseshoe bats – Evidence for an Acoustic Fovea. J. Comp. Physiol..

[27] Vater M, Feng AS, Betz M (1985). An HRP-study of the frequency-place map of the horseshoe bat cochlea: morphological correlates of the sharp tuning to a narrow frequency band. J. Comp. Physiol..

[28] Koessl M, Vater M (1985). The cochlear frequency map of the mustached bat, *Pteronotus parnelli*i. J. Comp. Physiol. A.

[29] Dannhof BJ, Bruns V (1991). The organ of Corti in the bat *Hipposideros bicolor*. Hear. Res..

[30] Schuller G, Beuter K, Schnitzler H-U (1974). Response to frequency shifted artificial echoes in the bat *Rhinolophus ferrumequinum*. J. Comp. Physiol..

[31] Suga N, Jen PH (1976). Disproportionate tonotopic representation for processing CF-FM sonar signals in the mustache bat auditory cortex. Science.

[32] Suga N, Neuweiler G, Möller J (1976). Peripheral auditory tuning for fine frequency analysis by the CF-FM bat. Rhinolophus ferrumequinum. J. Comp. Physiol..

[33] Rübsamen R, Neuweiler G, Sripathi K (1988). Comparative collicular tonopy in two bat species adapted to movement detection, *Hipposideros speoris* and *Megaderma lyra*. J. Comp. Physiol..

[34] Fu ZY, Tang J, Jen PHS, Chen QC (2010). The auditory response properties of single-on and double-on responders in the inferior colliculus of the leaf-nosed bat, *Hipposideros armiger*. Brain Res..

[35] Schnitzler H-UDD (1978). von Bewegungen durch Echoortung bei Fledermäusen. Verh. Dtsch. Zool. Ges..

[36] Schnitzler, H.-U. Echoes of fluttering insects: information for echolocating bats in *Recent advances in the study of bats* (eds Fenton, M. B., Racey, P. & Rayner, J. M. V.) 226–243 (Cambridge University Press, 1987).

[37] Schuller G (1979). Coding of small sinusoidal frequency and amplitude modulations in the inferior colliculus of ‘CF-FM’bat. Rhinolophus ferrumequinum. Exp. Brain Res..

[38] Neuweiler G, Bruns V, Schuller G (1980). Ears adapted for the detection of motion, or how echolocating bats have exploited the capacities of the mammalian auditory system. J. Acoust. Soc. Am..

[39] Schnitzler, H.-U. & Ostwald, J. Adaptations for the detection of fluttering insects by echolocation in horseshoe bats in *Advances in vertebrate neuroethology* (eds Ewert, J. P., Capranica, R. R. & Ingle, D. J.) 801–827 (New York: Plenum Press, 1983).

[40] Schuller G (1980). Hearing characteristics and Doppler shift compensation in south Indian CF-FM bats. J. Comp. Physiol..

[41] Gaioni SJ, Riquimaroux H, Suga N (1990). Biosonar behavior of mustached bats swung on a pendulum prior to cortical ablation. J. Neurophysiol..

[42] Henson OW, Koplas PA, Keating AW, Huffman RF, Henson MM (1990). Cochlear resonance in the mustached bat: behavioral adaptations. Hear. Res..

[43] Huffman RF, Henson OW (1993). Labile cochlear tuning in the mustached bat I. Concomitant shifts in biosonar emission frequency. J. Comp. Physiol. A.

[44] Hiryu S (2006). Intra-individual variation in the vocalized frequency of the Taiwanese leaf-nosed bat, *Hipposideros terasensis*, influenced by conspecific colony members. J. Comp. Physiol. A.

[45] Furusawa Y, Hiryu S, Kobayasi KI, Riquimaroux H (2012). Convergence of reference frequencies by multiple CF–FM bats (*Rhinolophus ferrumequinum nippon*) during paired flights evaluated with onboard microphones. J. Comp. Physiol. A.

[46] Liu Y, Feng J, Metzner W (2013). Different auditory feedback control for echolocation and communication in horseshoe bats. PloS one.

[47] Schnitzler H-UK (1967). von Dopplereffekten bei Hufeisen-fledermäusen. Naturwissenschaften.

[48] Schnitzler H-U (1970). Comparison of the echolocation behavior in *Rhinolophus ferrum-equinu*m and *Chilonycteris rubiginos*a. Bijdragen tot de Dierkunde.

[49] Konstantinov, A. I., Marakov, A. K. & Sokalov, B. V. Doppler-pulse sonar system in *Rhinolophus ferrumequinum* in *Proceedings of the Fourth International* Bat Research *Conference* (eds Olembo, R. J., Casteline, J. B. & Mutere, F. A.) 155–163 (Nairobi: Kenya National Academy for Advancement of Arts & Sciences, 1978).

[50] Jen PHS, Kamada T (1982). Analysis of orientation signals emitted by the CF-FM bat, *Pteronotus p*. *parnellii* and the FM bat, *Eptesicus fuscus* during avoidance of moving and stationary obstacles. J. Comp. Physiol..

[51] Lancaster WC, Keating AW, Henson OW (1992). Ultrasonic vocalizations of flying bats monitored by radiotelemetry. J. Exp. Biol..

[52] Hiryu S, Katsura K, Lin LK, Riquimaroux H, Watanabe Y (2005). Doppler-shift compensation in the Taiwanese leaf-nosed bat (*Hipposideros terasensis*) recorded with a telemetry microphone system during flight. J. Acoust. Soc. Am..

[53] Simmons JA (1974). Response of the Doppler echolocation system in the bat. Rhinolophus ferrumequinum. J. Acoust. Soc. Am..

[54] Henson (1982). Cochlear microphonic potentials elicited by biosonar signals in flying bats. Pteronotus p. parnellii. Hear. Res..

[55] Keating AW (1994). Doppler-shift compensation by the mustached bat: Quantitative data. J. Exp. Biol..

[56] Habersetzer J, Schuller G, Neuweiler G (1984). Foraging behavior and Doppler shift compensation in echolocating hipposiderid bats, *Hipposideros bicolor* and *Hipposideros speoris*. J. Comp. Physiol..

[57] Hiryu, S., Mora, E.C. & Riquimaroux, H. Behavioral and Physiological Bases for Doppler Shift Compensation by Echolocating Bats in *Bat* Bioacoustics (eds Popper, A. N. & Fay, R. R.) 239–263 (New York: Springer, 2016).

[58] Foeller E, Kössl M (2000). Mechanical adaptations for echolocation in the cochlea of the bat *Hipposideros lankadiva*. J. Comp. Physiol. A.

[59] Jen PHS, Suthers RA (1982). Responses of inferior collicular neurons to acoustic stimuli in certain FM and CF-FM Paleotropical bats. J. Comp. Physiol..

[60] Huffman RF, Henson OW (1991). Cochlear and CNS tonotopy: normal physiological shifts in the mustached bat. Hear. Res..

[61] Schuller G, Beuter K, Rübsamen R (1975). Dynamic properties of the compensation system for Doppler shifts in the bat. Rhinolophus ferrumequinum. J. Comp. Physiol..

