# Supplementary material for: Precise Doppler shift compensation in the hipposiderid bat, Hipposideros armiger
Source: Sci Rep. 2018 Mar 15;8:4598. doi: 10.1038/s41598-018-22880-y (PMC5854618; doi:10.1038/s41598-018-22880-y)
Supplement: Supplementary file 1 — Supplementary information [file 41598_2018_22880_MOESM1_ESM.pdf]

Precise Doppler shift compensation in the hipposiderid bat, *Hipposideros armiger*.

Diana Schoeppler[1*], Hans-Ulrich Schnitzler[1] and Annette Denzinger[1]

Authors affiliations:

[1] Animal Physiology, Institute for Neurobiology, University of Tübingen, Germany

*Corresponding author: Diana Schoeppler

Animal Physiology, Institute for Neurobiology, University of Tübingen,

Auf der Morgenstelle 28, 72076 Tübingen, Germany

Email: diana.schoeppler@uni-tuebingen.de

Supplementary information

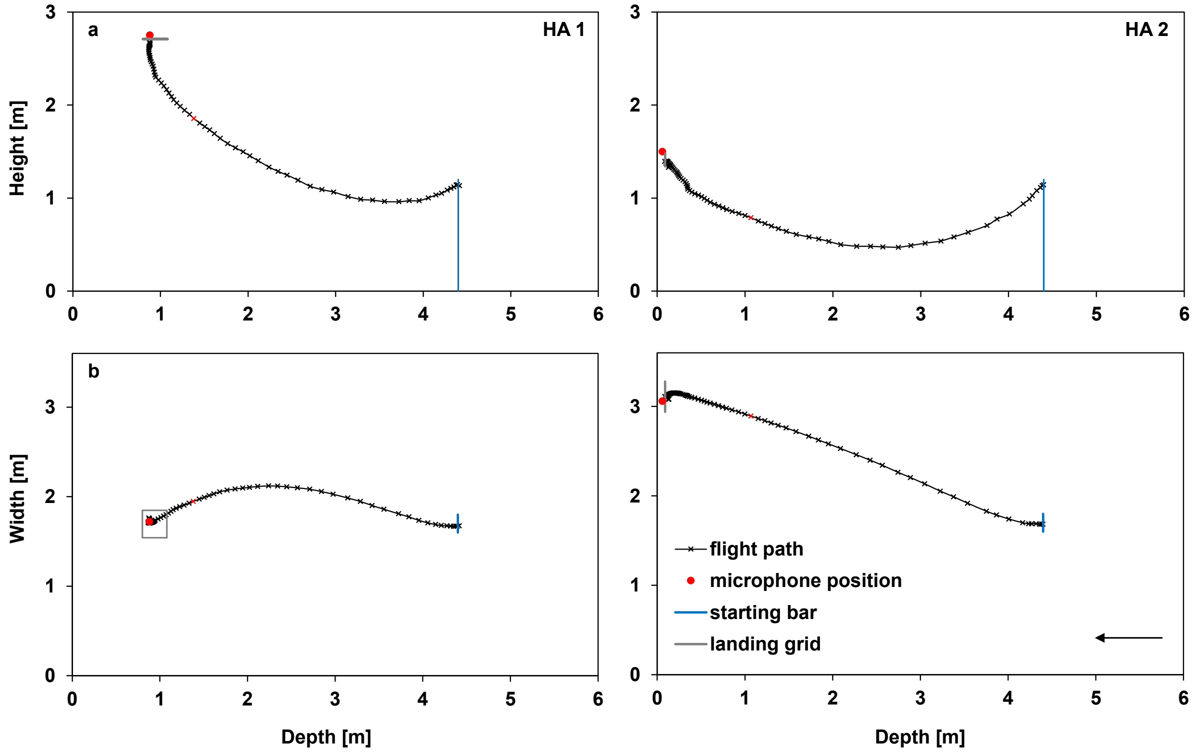

Figure S1: Flight path of bat 1 (HA 1) and bat 2 (HA 2). The lateral view is depicted in (a), the top view in (b), the arrow indicates the flight direction. Each emitted signal is marked with a cross, the first signal of the terminal approach with a red cross. The graphs display the size of the room in scale.
